# Melatonin, a Potential Therapeutic Agent for Preeclampsia, Reduces the Extrusion of Toxic Extracellular Vesicles from Preeclamptic Placentae

**DOI:** 10.3390/cells10081904

**Published:** 2021-07-27

**Authors:** Yunhui Tang, Katie Groom, Larry Chamley, Qi Chen

**Affiliations:** 1 Department of Family Planning, The Hospital of Obstetrics & Gynaecology, Fudan University, Shanghai 200081, China; 2 Department of Obstetrics and Gynaecology, The University of Auckland, 1142 Auckland, New Zealand; l.chamley@auckland.ac.nz; 3 National Women’s Health, Auckland City Hospital, 1142 Auckland, New Zealand; k.groom@auckland.ac.nz; 4 Liggins Institute, The University of Auckland, 1142 Auckland, New Zealand; 5 Hub for Extracellular Vesicle Investigations, The University of Auckland, 1142 Auckland, New Zealand

**Keywords:** melatonin, extracellular vesicle, exosome, preeclampsia, misfold proteins, endothelial cell activation

## Abstract

Preeclampsia, characterised by maternal endothelial cell activation, is triggered by toxic factors, such as placental extracellular vesicles (EVs) from a dysfunctional placenta. The increased oxidative stress seen in the preeclamptic placenta links to endoplasmic reticulum (ER) stress. The ER regulates protein folding and trafficking. When the ER is stressed, proteins are misfolded, and misfolded proteins are toxic. Misfolded proteins can be exported from cells, via EVs which target to other cells where the misfolded proteins may also be toxic. Melatonin is a hormone and antioxidant produced by the pineal gland and placenta. Levels of melatonin are reduced in preeclampsia. In this study we investigated whether melatonin treatment can change the nature of placental EVs that are released from a preeclamptic placenta. EVs were collected from preeclamptic (*n* = 6) and normotensive (*n* = 6) placental explants cultured in the presence or absence of melatonin for 18 h. Misfolded proteins were measured using a fluorescent compound, Thioflavin-T (ThT). Endothelial cells were exposed to placental EVs overnight. Endothelial cell activation was measured by the quantification of cell-surface ICAM-1 using a cell-based ELISA. EVs from preeclamptic placentae carried significantly (*p* < 0.001) more misfolded proteins than normotensive controls. Incubating preeclamptic placental explants in the presence of melatonin (1 µM and 10 µM) significantly (*p* < 0.001) reduced the misfolded proteins carried by EVs. Culturing endothelial cells in the presence of preeclamptic EVs significantly increased the expression of ICAM-1. This increased ICAM-1 expression was significantly reduced when the endothelial cells were exposed to preeclamptic EVs cultured in the presence of melatonin. This study demonstrates that melatonin reduces the amount of misfolded proteins carried by EVs from preeclamptic placentae and reduces the ability of these EVs to activate endothelial cells. Our study provides further preclinical support for the use of melatonin as a treatment for preeclampsia.

## 1. Introduction

Preeclampsia, a human specific pregnancy disorder, is clinically characterised by high blood pressure after 20 weeks of gestation accompanied by one or more of a spectrum of signs of organ dysfunction [1,2]. It affects 2–8% of all pregnancies worldwide [1,2]. The clinical signs of preeclampsia are preceded by an exaggerated inflammatory response and generalised maternal endothelial cell dysfunction, which are fundamental components of the pathogenesis of preeclampsia [3]. Dysfunctional endothelial cells consequently prevent the normal adaptation of the maternal vasculature seen in pregnancy, with resulting hypertension and other signs and symptoms of preeclampsia. Although the underlying mechanisms of preeclampsia are still not fully understood, it is recognised that one or more toxic factors released from the placenta trigger the maternal endothelial dysfunction. Extracellular vesicles (EVs) are among the placental factors that are increasingly recognised as being able to contribute to the endothelial cell dysfunction of preeclampsia [4,5,6].

EVs are lipid-enclosed packages of cellular contents that are extruded from all cells. Most eukaryote cells extrude both micro- and nano-EVs. While there are varying definitions, generally micro-EVs range in size from 200 to 1000 nm, while nano-EVs range from 10 to 200 nm. A subpopulation of nano-EVs are exosomes. The maternal-facing aspect of the human placenta is covered entirely by a single multinucleated cell, the syncytiotrophoblast, which extrudes vast numbers of both micro- and nano-EVs directly into the maternal circulation. These placental EVs are carried around the maternal body and can interact with multiple maternal cells and organs, including endothelial cells [7,8]. Like EVs from other cell types, placental EVs contain biologically active proteins, nucleic acids, lipids and RNAs, that they can transfer to recipient cells and organs, affecting their function [9,10,11,12]. We and others have previously reported that placental EVs derived from preeclamptic placentae activated maternal endothelial cells in vitro and shown that nano-EVs alter the maternal vascular tone in pregnant mice [10,13,14,15].

Oxidative stress is significantly increased in the preeclamptic placenta [16], and there is a strong connection between oxidative stress and endoplasmic reticulum (ER) stress [17]. The ER regulates protein folding and trafficking. When the ER is stressed, proteins are misfolded, and misfolded proteins can then be exported from cells or tissues, via EVs which target to other cells where the misfolded proteins may be toxic. We have recently shown that preeclamptic placentae contained significantly more aggregated transthyretin than normotensive placentae and that this transthyretin is specifically packaged into nano-EVs [18].

Melatonin is a lipid-soluble hormone with antioxidant activities originally identified as being of primarily pineal origin. However, melatonin is also produced in large quantities by the placenta [19,20]. In addition to its endocrine functions, melatonin also has direct free radical scavenging and indirect antioxidant activities [21,22,23,24]. The peak melatonin levels in pregnancy are 80–100 pg/mL, twice as high as those in men or non-pregnant women. Much of the melatonin is of placental origin, suggesting a particular importance of melatonin in pregnancy [19,25,26,27,28]. However, women with preeclampsia have significantly decreased serum levels of melatonin [25,26], as well as decreased levels of the enzymes responsible for melatonin synthesis and decreased levels of melatonin receptors [29]. Given the action of melatonin in modulating free radicals/oxidative stress and a potential role in the treatment of preeclampsia [30,31,32], we undertook this study to investigate whether melatonin supplementation has a preventative effect on the production of toxic placental EVs from preeclamptic placentae.

## 2. Methods

This study was approved by the Northern X Health and Disabilities Ethics Committee, New Zealand (NTX/12/06/057/AM06), and conforms to the principles outlined in the Declaration of Helsinki. All patient-derived tissues were obtained following informed written consent.

## 3. Collection of Preeclamptic Placentae

Six placentae were collected from women with preeclampsia (two of them in an early onset and four of them in a late onset form), and ten term placentae were collected from normotensive pregnancies from National Women’s Health, Auckland City Hospital, New Zealand. The clinical parameters of the study cohort are summarised in Table 1. We were not able to collect gestation-matched placentae from normotensive pregnancies due to ethical issues. There was no difference in the maternal age (*p* = 0.388) between the two groups. All women with preeclampsia received medication, either labetalol or nifedipine. The mean BMI in preeclampsia was 27.08 ± 5.2 kg/m^2^.

Preeclampsia was defined as a maternal systolic blood pressure ≥140 mmHg and/or diastolic blood pressure ≥90 mmHg on two occasions separated by 6 h, and proteinuria >300 mg in a 24 h period, a protein-creatinine ratio >30 mg/mmol or impaired liver function after 20 weeks of gestation, in accordance with the guideline of the Society of Obstetric Medicine of Australia and New Zealand (SOMANZ), which are consistent with the international guideline from the International Society for the Study of Hypertension in Pregnancy (ISSHP) [33].

## 4. Collection of Placental EVs from Preeclamptic Placentae

Approximately 400 mg wet weight placental explants were dissected from either preeclamptic or normotensive placentae, as previously described [13,34,35]. Four quadruplicate explants from each placenta were cultured in Netwell™ culture inserts and suspended in 12 well culture plates, for 24 h at 37 °C, in 3 mL Advanced DMEM/F12 containing 2.5% fetal bovine serum in an ambient oxygen atmosphere in the presence or absence of melatonin (1 µM and 10 µM, dissolved in PBS) (Sigma-Aldrich, Auckland, New Zealand). The concentration of melatonin followed that of our previous study [36]. As our previous study [36] showed no dose response on the effect of melatonin, in this study normotensive placental explants were treated with a higher dose of melatonin (10 µM). The conditioned media were then collected and centrifuged at 2000× *g* for 5 min for the removal of culture debris. The supernatant was centrifuged at 20,000× *g* for 1 h for the collection of micro-EVs. The supernatant was further centrifuged at 100,000× *g* for 1 h for collection of nano-EVs (Avanti J30 I Ultracentrifuge, JA 30.50 fixed angle rotor, Beckman Coulter, New Zealand).

The relevant cultured explants with melatonin treatment were then collected and immediately embedded in an optimal cutting temperature (OCT) compound, and then sectioned at 5 µm using a cryostat for the measurement of misfolded proteins.

## 5. Quantification of Micro-EVs and Nano-EVs

The amount of micro- and nano-EVs collected from preeclamptic and healthy placentae were quantified using a Nanosight™ NS300 nanoparticle tracking device (Nanosight, UK). Placental micro-EVs and nano-EVs were re-suspended in 1 mL PBS after ultra-centrifugation and analysed. All automatic settings were applied, with the viscosity setting at 0.95 cP and the temperature at 25 °C. A single measurement consists of three 30-s videos, and each sample was measured five times at camera level 10. The detection threshold was set at 10, and data acquisition and processing were performed using the NTA3.2 software (Nanosight). Only recordings with over 1000 valid tracks/vesicles were included in the analysis. The number of placental EVs was expressed as the number of EVs per mL.

## 6. Measurement of Endothelial Cell Activation by Cell-Based ELISA

A human microvascular endothelial cell line, composed of HMEC-1 cells (ATCC, CRL-3243), was grown until confluent in 96 well culture plates in MCDB 131 media. The endothelial cells (HMEC-1) were exposed to placental micro- or nano-EVs from preeclamptic placental explants or from normotensive placental explants which had been treated with or without melatonin for 24 h. After the removal of the remaining placental EVs by washing with PBS, the cell surface expression of ICAM-1 by HMEC-1 monolayers was determined by a cell-based ELISA, as described previously [13]. Each measurement of ICAM-1 was conducted in quadruplicate. The data are presented as the median and 5th and 95th percentiles of the fold changes relative to untreated controls.

## 7. Measurement of Misfolded Proteins

Misfolded proteins in preeclamptic and healthy placentae and in the preeclamptic placental explants that had been treated with melatonin were measured using a fluorescent compound, Thioflavin-T (ThT, Sigma-Aldrich, Sydney, Australia), as described previously [37,38]. Briefly, frozen placental sections (5 µm) were fixed with 4% paraformaldehyde (PFA) for 5 min at room temperature and washed with PBS. The sections were then stained with ThT (500 µM dissolved in PBS) for 3 min at room temperature, followed by counter-staining with DAPI for 1 min. The sections were then mounted with Citifluor and examined using a fluorescent microscope (Nikon, ECLISPE Ni-E, Tokyo, Japan). Sections from which ThT was omitted were used as negative controls.

Misfolded proteins in micro- or nano-EVs collected from preeclamptic or healthy placental explant cultures that had been treated with melatonin were stained with ThT (5 µM) for 10 min and read in a fluorescent plate reader at 485 nm (Synergie 2, BioTek, Auckland, New Zealand), following previous reports [37,38].

## 8. Semiquantitative Analysis of Immunofluorescent Staining

All images were converted into a 32-bit RGB colour format. Subsequently, a representative mean grey value integral was quantified from each image, which served as an index for fluorescence intensity and allowed for relative comparisons of protein expression between different images and samples. Each image was opened using ImageJ software, and five lines were randomly drawn on the images. The fluorescent density of each line on the images was calculated by Matlab software (R2019 A version). The resultant five integral values were averaged to yield a mean grey value integral [39].

## 9. Statistical Analysis

The concentrations of placental micro- and nano-EVs were expressed as a median and 95% confidence interval (CI). The statistical analysis of the concentration of placental micro- or nano-EVs, the levels of expression of cell surface ICAM-1 by endothelial cells or the levels of misfolded proteins in placental EVs were assessed with an ANOVA test, or a Mann–Whitney U-test, when appropriate. The GraphPad Prism software package (version 8.0) was used, and a *p* < 0.05 was considered as statistically significant.

## 10. Results

### 10.1. The Amount of Placental EVs Extruded from Preeclamptic Placentae Was Not Changed by Treatment with Melatonin

**Figure 1:**
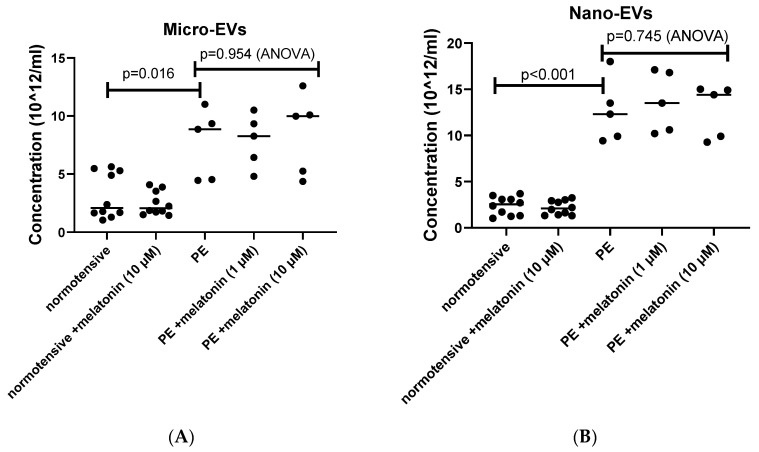
The numbers of micro- (**A**) or nano-EVs (**B**) extruded from preeclamptic placentae (PE) was quantified by NTA, and the statistical analyses were assessed with an ANOVA test or a Mann–Whitney U-test, when appropriate.

We confirmed previous reports [40] that the amount of micro- and nano-EVs extruded from preeclamptic placentae was significantly increased compared to the number of EVs released from normotensive placental explants (Figure 1A, *p* = 0.016, or Figure 1B, *p* < 0.01, respectively). The treatment with melatonin (either 1 µM or10 µM) did not change the amount of micro-EVs (Figure 1A, *p* = 0.954, ANOVA) or nano-EVs (Figure 1B, *p* = 0.776, ANOVA) extruded from preeclamptic placental explant cultures.

### 10.2. Treatment with Melatonin Prevented the Production of Toxic Placental EVs from Preeclamptic Placentae

**Figure 2:**
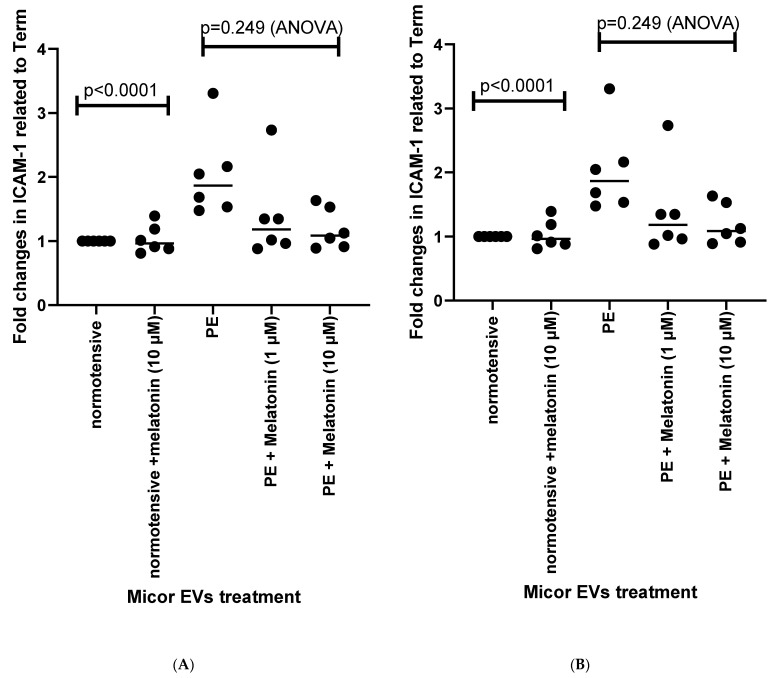
The activation of endothelial cells after being exposed to micro- (**A**) or nano-EVs (**B**) extruded from preeclamptic (PE) or normotensive placentae was measured by cell-surface ICAM-1 levels. The statistical analysis was assessed by an ANOVA test or a Mann–Whitney U-test, when appropriate.

We confirmed that placental EVs from preeclamptic placentae activated endothelial cells (Figure 2A,B, *p* < 0.001), which was previously reported [41]. Both the micro-EVs and nano-EVs released from preeclamptic placental explants that had been incubated with melatonin did not induce an increase in endothelial cell activation (as measured by cell surface ICAM-1 expression, Figure 2A, *p* = 0.0014, ANOVA or Figure 2B, *p* = 0.0002, ANOVA). There was no dose response in the reduction of ICAM-1 levels (*p* > 0.05) across the concentrations of melatonin tested.

### 10.3. Treatment with Melatonin Reduced the Levels of Misfolded Proteins in Preeclamptic Placentae and in Placental EVs from Preeclamptic Placentae

**Figure 3:**
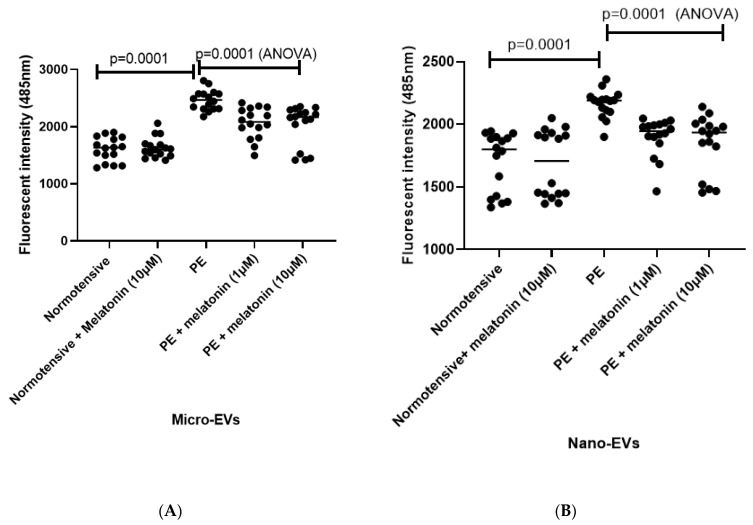
The misfolded proteins in placental micro-EVs (**A**) and nano-EVs (**B**) collected from either preeclamptic (PE) or normotensive placentae were measured by ThT fluorescent intensity. The statistical analysis was assessed by an ANOVA test or a Mann–Whitney U-test, when appropriate.

There were significantly increased levels of misfolded proteins in micro- (Figure 3A, *p* = 0.0001) and nano-EVs (Figure 3B, *p* = 0.0001) collected from preeclamptic placentae compared to placental EVs collected from normotensive term placental explants, measured by the fluorescent intensity of ThT (measured at 485 nm). However, this increased fluorescent intensity of ThT was significantly reduced when placental EVs were collected from melatonin-treated (1 or 10 µM) preeclamptic placental explant cultures (Figure 3A,B, *p* = 0.0001, ANOVA). There was no dose response in the reduction of the fluorescent intensity of ThT.

**Figure 4:**
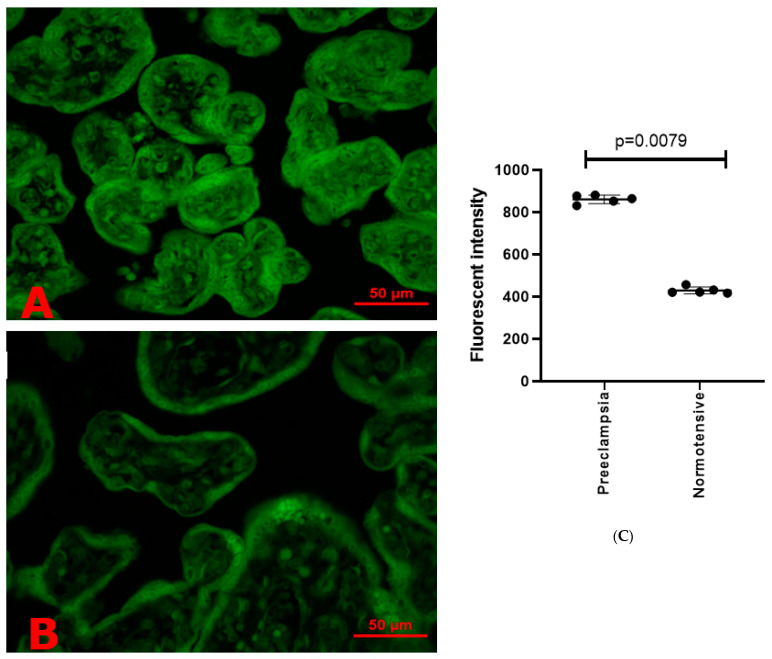
The levels of misfolded proteins in preeclamptic placentae, especially in the syncytiotrophoblast (**A**) and in normotensive term placentae (**B**), were measured by ThT fluorescent intensity. The statistical difference was confirmed by a semi-quantitative assay (**C**).

**Figure 5:**
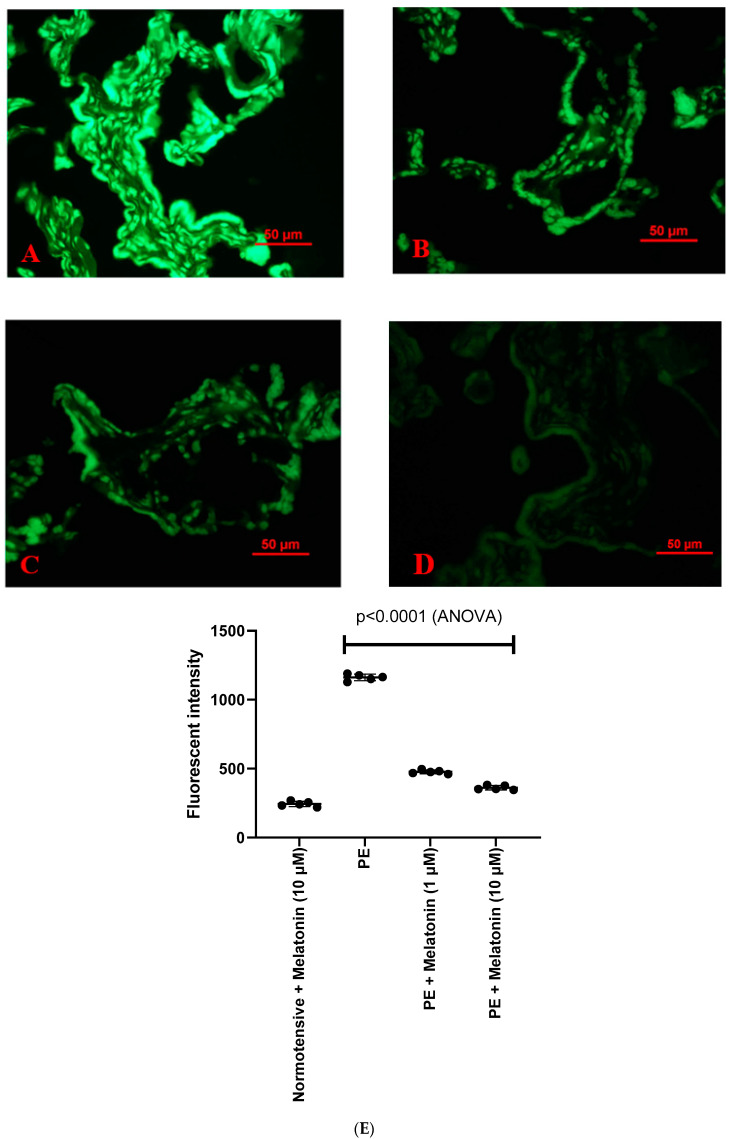
Misfolded proteins in preeclamptic placental explants (PE) that had been treated with 1 µM (**B**) or 10 µM (**C**) melatonin or without melatonin (**A**) were measured by the fluorescent intensity of ThT. In comparison, misfold proteins in normotensive placental explants that had been treated with 10 µM (**D**) were also measured. The statistical difference was confirmed by a semi-quantitative assessment and assessed by an ANOVA test (**E**).

In addition, there were significantly increased levels of misfolded proteins in preeclamptic placentae (Figure 4A), compared to normotensive placentae (Figure 4B), measured by the increased fluorescent intensity levels of ThT in a semi-quantitative assay (Figure 4C, *p* = 0.0079). However, the treatment of the preeclamptic placentae with melatonin (1 µM Figure 5B or 10 µM Figure 5C) significantly reduced the fluorescent intensity of ThT in preeclamptic placentae, compared to the untreated ones (Figure 5A), measured by a semi-quantitative assay (Figure 5E, *p* < 0.0001). There was also no dose response in the reduction of the fluorescent intensity of ThT (Figure 5E, *p* > 0.05).

## 11. Discussion

In this in vitro study, we found that the treatment with melatonin did not reduce the amount of EVs produced by preeclamptic placentae but did alter the nature (function) of the EVs such that they were not toxic and did not active endothelial cells. Reducing the increased levels of ER stress in preeclamptic placentae and in EVs that were exported from preeclamptic placentae could be one of the underlying mechanisms of this protective effect of endothelial cell activation.

Although the exact causes of preeclampsia are still unclear, there is increasing evidence suggesting that EVs extruded from the placenta are associated with the pathogenesis of preeclampsia [13]. These placental EVs from preeclamptic placentae may contribute to triggering the disease by inducing maternal systemic endothelial cell dysfunction [42], as they carry several anti-angiogenic factors, including soluble fms-like tyrosine kinase-1 (sFlt1) and soluble Endoglin (sEng), cytokines and oxidants, as well as multiple “danger” signals [43]. Oxidative stress results from an imbalance in oxidant and antioxidant molecules [44] and is reported to play an important role in the development of preeclampsia [45].

Melatonin, typically thought of as a lipid hormone that regulates circadian rhythms, has been shown to have direct free-radical scavenging properties and to induce the expression of antioxidants [46]. During pregnancy, the placenta is a major source of melatonin, producing higher levels than the pineal gland [20], and placental melatonin production does not appear to be regulated in a circadian fashion [47]. Studies have suggested that alterations of circadian rhythms increase the risk of developing preeclampsia [48]. In women with preeclampsia there are significantly reduced levels of circulating melatonin [25,26], accompanied by a reduction in the levels of the synthetic enzymes and melatonin receptors in preeclamptic placentae [29]. Therefore, it has been suggested that exogenous melatonin may have potential benefits in preeclampsia [30,31,32,48,49]. Here, we confirmed that EVs from preeclamptic placentae induce endothelial cell activation, which is a hallmark of preeclampsia, and found that EVs from preeclamptic placental explants that had been treated with melatonin did not induce endothelial cell activation. This finding fits with our previous report that melatonin reversed the production of toxic placental EVs extruded from normal placental explants that had been treated with either preeclamptic serum or antiphospholipid antibodies, which are a major maternal risk factor for preeclampsia [50]. In our previous report, we demonstrated that treating placental explants with melatonin reduced the markers of oxidative stress that were induced by the preeclamptic sera and antiphospholipid antibodies [36]. Others have recently shown that melatonin induces the expression of antioxidants in the placenta [51]. Taken together, our data further suggest that exogenous melatonin could directly change the production of toxic EVs by the preeclamptic placenta.

The molecular signalling pathways that change the production of EVs in preeclampsia are largely unknown. A number of factors found in preeclampsia, such as the inflammatory cytokines IL-6 and TNFα [52], disrupt placental function, resulting in the production of toxic EVs [53]. Exactly how those cytokines induce the production of toxic EVs is still unclear, but more is known about how antiphospholipid antibodies induce the production of toxic EVs [54,55]. These autoantibodies are internalised into the syncytiotrophoblast where they cause mitochondrial dysfunction, oxidative damage, the production of both necroptosis and apoptosis cell death pathway proteins and ER stress, characterised by an excess of misfolded proteins [38,56]. ER stress is a feature of preeclamptic placentae that is characterised by increased protein misfolding and aggregation (reviewed in [57]). These misfolded and aggregated proteins are toxic and can be deported from the placenta into the maternal circulation via EVs. For example, we have previously shown increased levels of aggregated transthyretin in EVs extruded from preeclamptic placentae [18]. Removing misfolded proteins via EVs may be a survival mechanism for the syncytiotrophoblast, but these misfolded proteins are potentially damaging to the maternal cells that take up the EVs containing an excess of misfolded proteins. Here, we confirmed an increased level of misfolded proteins in preeclamptic placentae. In addition, we also found an increased level of misfolded proteins in both micro- and nano-EVs extruded from preeclamptic placentae. These EVs are targeted to several maternal organs, such as the liver and kidneys, that are often effected in preeclampsia [9,10]. The uptake of micro-EVs and/or nano-EVs that contain toxic misfolded proteins may be harmful to these organs. The key finding of our current study is that treating explants from preeclamptic placentae with melatonin reduces the levels of misfolded proteins both in the placenta and in the EVs extruded from the placentae. The reduction of the load of misfolded proteins delivered to maternal organs by EVs from melatonin-treated preeclamptic placentae may reduce the harm to those organs. The clinical utility of melatonin for the treatment of preeclampsia has recently been suggested by a phase I clinical trial that demonstrated the safety of administering melatonin to women with preeclampsia and also demonstrated a prolongation of the time from diagnosis to delivery [58].

We acknowledge that, due to the small clinical samples used in this study, we were not able to analyse whether the protective effect on producing toxic placental EVs by melatonin is associated with the severity or time of onset of preeclampsia. Future study is required to confirm our findings.

In conclusion: this study demonstrates that exogenous melatonin prevents the production of endothelial-activating EVs from preeclamptic placentae. Reducing the amount of misfolded proteins carried by EVs from preeclamptic placentae exporting to the maternal circulation may be one of the underlying mechanisms to prevent endothelial cell activation. Our study provides further preclinical support for the use of melatonin for the treatment of preeclampsia.

## Figures and Tables

**Table 1:** Clinical parameters of the study cohort.

	Preeclampsia (*n* = 6)	Normotensive (*n* = 10)
Maternal age (years, mean/SD)	30.2 ± 3.1	32 ± 5.1
Onset week (mean/SD)	33 + 6 ± 4	N/A
Delivery week	35 + 2 ± 4	39 ± 1
Birthweight (g, mean/SD)	2365 ± 338	3350 ± 180
Systolic blood pressure (mmHg, mean/SD)	156 ± 7	N/A
Diastolic blood pressure (mmHg, mean/SD)	98 ± 11	N/A

## Data Availability

All the data will be available upon to request.
